# Autonomous Incident Detection on Spectrometers Using Deep Convolutional Models

**DOI:** 10.3390/s22010160

**Published:** 2021-12-27

**Authors:** Xuelin Zhang, Donghao Zhang, Alexander Leye, Adrian Scott, Luke Visser, Zongyuan Ge, Paul Bonnington

**Affiliations:** 1 Monash eResearch Centre, 15 Innovation Walk, Monash University, Clayton Campus Victoria, Clayton, VIC 3800, Australia; xuelin.zhang@monash.edu (X.Z.); donghao.zhang@monash.edu (D.Z.); anley1@student.monash.edu (A.L.); 2 Agilent Technologies, 679 Springvale Rd, Mulgrave, VIC 3170, Australia; adrian.scott@agilent.com (A.S.); luke.visser@agilent.com (L.V.)

**Keywords:** machine vision, deep learning, object detection

## Abstract

This paper focuses on improving the performance of scientific instrumentation that uses glass spray chambers for sample introduction, such as spectrometers, which are widely used in analytical chemistry, by detecting incidents using deep convolutional models. The performance of these instruments can be affected by the quality of the introduction of the sample into the spray chamber. Among the indicators of poor quality sample introduction are two primary incidents: The formation of liquid beads on the surface of the spray chamber, and flooding at the bottom of the spray chamber. Detecting such events autonomously as they occur can assist with improving the overall operational accuracy and efficacy of the chemical analysis, and avoid severe incidents such as malfunction and instrument damage. In contrast to objects commonly seen in the real world, beading and flooding detection are more challenging since they are of significantly small size and transparent. Furthermore, the non-rigid property increases the difficulty of the detection of these incidents, as such that existing deep-learning-based object detection frameworks are prone to fail for this task. There is no former work that uses computer vision to detect these incidents in the chemistry industry. In this work, we propose two frameworks for the detection task of these two incidents, which not only leverage the modern deep learning architectures but also integrate with expert knowledge of the problems. Specifically, the proposed networks first localize the regions of interest where the incidents are most likely generated and then refine these incident outputs. The use of data augmentation and synthesis, and choice of negative sampling in training, allows for a large increase in accuracy while remaining a real-time system for inference. In the data collected from our laboratory, our method surpasses widely used object detection baselines and can correctly detect 95% of the beads and 98% of the flooding. At the same time, out method can process four frames per second and is able to be implemented in real time.

## 1. Introduction

Machine vision, also known as industrial computer vision, is the key component of Industry 4.0 for the industrial automation revolution. It joins machine learning and computer vision in a set of tools that are able to grant industry-level instruments unprecedented abilities to observe and interpret their environment [1]. Industrial activities involving machine vision systems such as incident detection, product quality monitoring, and manufacturing automation, have demonstrated tremendous potential for bolstering productivity, reducing waste, refining product quality, enhancing manufacturing flexibility, and decreasing operating costs. Machine vision plays an essential role in bolstering productivity for modern precise agriculture, which is challenging due to wind disturbance, changing illumination, and object occlusion. Some examples include using object detection and 3D mapping to allow robots harvest fruits automatically in plant industries [2,3].

**Figure 1:**
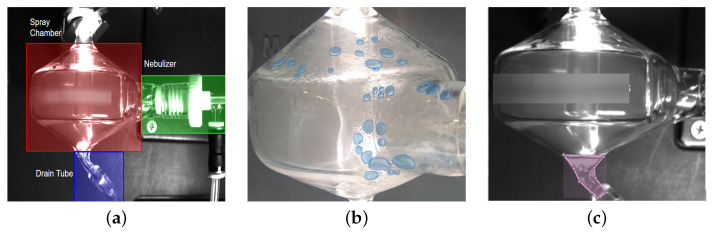
(**a**) Region of interest from a typical spectrometer device where most incidents happen. It contains a spray chamber, a nebulizer, and a drain tube connected to the chamber. (**b**) Beads on the surface of the spray chamber. This is the first type of incident to be detected. In the image, the beads are labeled with instance masks. (**c**) Flooding at the bottom of the spray chamber. This is the second type of incident to be detected. The flooding area is indicated with a pink color.

Incident detection, which is one of the most longstanding and fundamental problems in industrial computer vision, has attracted great attention consistently in the past several decades since it plays an important role in high-cost and safety-critical industrial activities. For example, Erhu et al. [4] introduced an automatic defect detection system for web offset printing based on modern machine vision. The detection system has a sensitivity of 94.86% and can process seven images per second. Chengyu et al. [5] proposed abnormal event detection system for bolts looseness detection in flange connection. Bartosz et al. [6] introduced a quality control automation of electric cables, which is able to detect defects in electric cables. Chen et al. summarizes data-driven fault diagnosis methods that can detect and diagnose faults for traction systems in high-speed trains automatically. [7] While existing incident detection works have achieved tremendous success in incident detection in terms of the manufacturing process, applying them to other industrial scenarios such as the chemical industry is still a large challenge because objects to be tested have totally different properties. For example, spectrometers widely used in chemistry analytical laboratories for chemical composition analysis can suffer from spray chamber contamination causing an increase in surface tension of the glass resulting in a liquid beading phenomenon or when drainage fails to occur resulting in the flooding of the chamber. The former can cause erroneous analytical results and the latter can cause torch fountaining (spray of the sample, often acidic) into the instrument potentially damaging it. Figure 1a shows an exemplar spectrometer. The vaporized chemical substances enter through the nebulizer into the spray chamber and then be aspirated on the top of this chamber for spectrum analysis. The vaporized chemical substances which are not successfully aspirated will be condensed to generate beads. These chemical substances can also cause flooding if drainage does not occur.

Our aim is to detect bead and flooding incidents, which can be seen in the Figure 1b and Figure 1c respectively. These incidents can appear due to the wrong setup of devices and can result in wrong results obtained from the spectrometers. The current solution to prevent these incidents from happening is to assign a specialist to monitor the device all the time, which is both time-consuming and labor-intensive.

However, detecting these incidents is challenging. As can be seen in Figure 1, both beads and flooding are liquids, which are transparent and deformable. At the same time, beads are small in size. So we need a framework to detect small and transparent objects. Small object detection is always a challenging problem. Existing object detection methods usually have a lower detection accuracy on small objects when compared to that of large objects since small objects lack sufficient detailed appearance information. At the same time, beads are also transparent. Transparent objects unavoidably introduce difficulties and ambiguities to confuse state-of-the-art object detectors due to varying features. The shape of flooding depends on its container. All of these make accurate detection of incidents challenging. As a result, there is no related existing incident detection methods in similar industry that can detect beads or flooding.

In this work, we propose the novel incident detection system of spectrometers for chemical industry. The incident detection system is capable of detecting two types of incidents occuring in spectrometers: flooding and beads. The flooding detection system is composed of two parts. The first part is a Mask RCNN [8] model. The model predicts bounding boxes for flooding and drain tube in an image. The second part is a temporal model. To detect the deformable flooding, we use time series data and the relationship between flooding and bubbles as clues to improve the accuracy of flooding detection. In details, the confidence scores of the flooding bounding boxes and the difference between drain tube regions of two consecutive images are calculated and used as the input to the temporal model.

For bead detection, we experiment with different components, including the backbone and the detector architecture, of object detection models. After extensive experiments, we choose HRNetVV2p [9] as the backbone and Hybrid Task Cascade [10] (HTC) as the detector. To furthermore boost the performance and robustness of our model, we use Online Hard Example Mining (OHEM) sampler [11] and Cut-Paste augmentation [12]. It is difficult and challenging to detect and label small and transparent objects, so the cut-paste augmentation is implemented to increase the robustness of model to detect small changes in the pattern and avoid the labor-intensive labelling process.

Since there is no existing data available for our task, we have built our own dataset using images or videos collected from a chemistry lab. The data is collected under a controlled environment, which is the same as the real scenarios since experiments using spectrometers are run in chemistry labs. As mentioned in [13], there are three main components in a incident detection system, the light source, the detected object, and the camera. The detected objects are the beads and flooding and are manually created by running the spectrometer and are the same as in real world scenarios. The main light source is the light above the spray chamber and is part of the device. To improve the robustness of our method, we have also collected images under different illumination conditions. Finally, we set the location of the camera to be in front of the spray chamber, which is a practical location of the camera under real-world circumstances. To further improve the robustness of our method and look for other candidate positions for the camera, we collect some images from different angles. All data are collected in real scenarios and the spectrometers are operated by chemistry technicians to ensure their realness.

Our contributions are summarized as follows:

1. In this paper, we proposed the novel industry computer vision system for incident detection of spectrometers. The developed framework can detect 95% of the beads and 98% of the flooding under the controlled lab environment and is able to process four frames per second, which is fast enough to be implemented in real-time.
2. Since there is no method with high accuracy to detect transparent and deformable flooding, based on our observation of the relationship between flooding and bubbles in the drain tube region, we convert the hard flooding detection task into a simple bubble movement detection task and propose to use a temporal model with low computation cost. We calculate the pixel differences of the drain tube region of two consecutive images to include temporal information. Then we input a sequence of pixel differences along with the confidences scores of the flooding detection bounding boxes to a basic neural network to predict the existence of floodings. This allows our pipeline to give temporal consistent predictions of flooding as well as preventing the heavy computation cost of using convolutional neural network with recurrent neural network.
3. Targeting at the challenges and difficulties introduced by the non-rigid properties of bead objects, we select the best combination of object detection model components. To tackle with the small amount of data and annotations we have, data synthesis and augmentation are integrated into the proposed bead detection framework. Furthermore, the hard negative mining sampler is to guide our model to learn to detect more beads accurately. Our bead detection model outperforms state-of-the-art object detectors.

## 2. Related Work

### 2.1. Object Detection Models

An object detection model contains two parts. The first part is a backbone pretrained on a large object detection dataset, such as ImageNet [14] and common objects in context (COCO) [15]. And the second part is a head classifier and localizer to predict the classes and bounding box locations of the objects. Object detection models can be split into two categories, two-stage detection models and one-stage detection models. In two-stage detection models, the backbone extracts features and then generates region proposals. Then, the regions of interest is processed by the classifier heads. Some representative detectors in this class are RCNN [16], Fast RCNN [17], Faster RCNN [18], Mask RCNN [8], and Libra RCNN [19]. On the other hand, one-stage detection models are end-to-end and don’t have the region proposal step. Some examples are OverFeat [20], YOLO [21], SDD [22], CornerNet [23], and CenterNet [24]. Mask RCNN is adapted in the first stage of flooding detection since Mask RCNN is a state-of-the-art baseline for object detection tasks.

### 2.2. Small Object Detection

One problem of object detection models is the low accuracy on small objects. Some models use attention and context information to mitigate this problem [25,26]. Other models use multi-scale features. In the past, object detection models usually use image pyramid as a method to get multi-scale features and only used at inference. Then in 2016, Feature Pyramid Network (FPN) [27] is proposed, which consists of both bottom-up pathway and the top-down pathway. It first builds a bottom-up pathway where the feature maps become smaller and smaller. Then the top-down pathway is built using the feature map in the corresponding layer and the up-sampled feature map in the same path way. By using these two pathways to explicitly address multi-scale problems, a model can achieve better accuracy. Similar to FPN, YOLOv3 [28] predicts boxes at 3 different scales and feature maps with different resolutions are used to make the final prediction. There are many variations of FPN, such as PANet [29], FPR [30], DetNet [31], and M2Det [32]. In our work, we use FPN in the backbone for the Mask RCNN model.

### 2.3. Transparent Object Detection

Transparent object detection is another challenging problem. It is extremely difficult because transparent objects lack information of textures, their appearances depend on the environments, and their edges are blurred and implicit [33,34,35]. There are few models designed specifically to solve this challenge. Po-Jen and Chiou-Shann [34] uses RCNN with selective search [36]. They improve the efficiency of the model by using characteristics of transparent objects to improve filter the RoIs generated by selective search. We do not use this method because this method improves the precision of transparent object detection but does not improve the recall. Other methods [33,35] estimate the depth of transparent objects with RGB-D information. The same principle can not be applied here as the beads and flooding to be detected are all inside the container, meaning they have nearly the same depth. Furthermore, in all the methods mentioned above, the transparent objects to be detected are glasses, which have more texture than liquids and have consistent shapes. It turns out that we can not simply apply existing methods to tackle problems of flooding detection and bead detection.

## 3. Methodology

**Figure 2:**
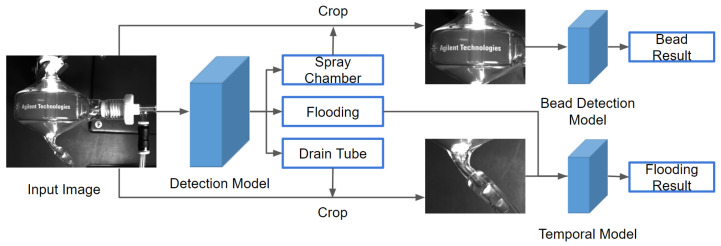
The whole pipeline of our method. We use an object detection model to obtain bounding boxes of the spray chamber, the drain tube, and flooding in an input image. Then we crop the spray chamber region and the drain tube region. Another detection model is applied to the spray chamber region for bead detection. We use a temporal model on a series of drain tube region images and confidence scores of the flooding bounding box to detect flooding.

We designed two malfunction detection frameworks relating to computer vision and object detection to identify the flooding of spray chamber and bead detection. The visual phenomenon of flooding is the accumulation of liquids beyond the safe level. The occurrence of flooding is a sequential event with upper liquid surface in the spary chamber increasing gradually. The beads on the surface of a spray chamber are transparent and deformable. The number, size and regions of beads provide useful information to detect the malfunction. The appearance of flooding and beads may disrupt the spray mist flow pattern and may result in an inaccurate spectrum analysis. The whole pipeline of our method is shown in Figure 2.

### 3.1. Flooding Detection

**Figure 3:**
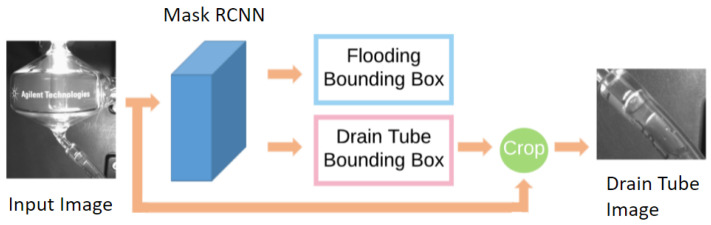
The Mask RCNN stage of the flooding detection. First, we use Mask RCNN to generate bounding boxes for flooding and the drain tube. Then we crop the drain tube region from the input image. The drain tube image and the flooding bounding box enter the next stage of flooding detection.

**Figure 4:**
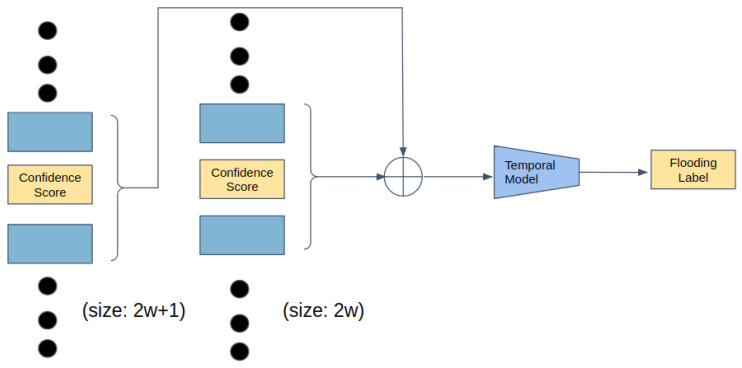
The temporal model stage of the flooding detection. We define a window size *w*. Then for an image at time *t*, we concatenate the confidence scores of flooding bounding box of images from time t−w to t+w, and the mean squared error (MSE) of the pixel values between two consecutive drain tube images. We input them into the temporal model to predict the existence of flooding at time *t*.

Flooding detection means detecting the accumulation of liquids at the bottom of the spray chamber in Figure 1c. It is a challenging problem because flooding is transparent and deformable. This means it lacks textures and its shape depends on its container. To solve the problem, we propose the flooding detection model. The flooding detection model includes two parts. The first part is a Mask RCNN model for detecting the flooding and the drain tube region Figure 1a, as can be seen in Figure 3. The second stage is the post-processing stage which uses time series data to improve the accuracy and consistency, as can be seen in Figure 4. By adding temporal information at the second stage, the flooding detection model can achieve a high accuracy while avoiding a high overhead.

**Stage 1: Mask-RCNN** Mask RCNN is a two-stage model. The first stage is called Region Proposal Network (RPN) [18], which proposes candidate bounding boxes. The second stage is extracting features from the proposed regions, using RoIAlign layer [8] to combine them and a classifier to predict the classes. There is also a mask branch to generate masks of the objects.

Our framework uses Mask RCNN without the masking branch as the first stage. The reason to use this instead of Faster RCNN is that the RoIAglin layer proves to improve the accuracy. And since flooding is deformable, its shape is based on the container. When training with the masking branch, the model appears to predict the bottom part of the spray chamber instead of flooding. For this reason, we removed the masking branch. We use the default hyper-parameters following the existing Mask RCNN work. The threshold of RoI score is set to 0.5 such that a bounding box with a class score higher than 0.5 is positive, and negative otherwise. The images are scaled to the same size after entering the model by default. Each mini-batch has 2 images per GPU. The backbone used is ResNet101 [37] pretrained on ImageNet. FPN is also used in the model. The model is trained for 30 epochs with 50 steps per epoch. Since there can only be one flooding in each image, only the bounding box with the highest confidence score is kept. The output of the Mask RCNN are the confidence score $S_{f}$ of the flooding bounding box, ranging from 0 to 1 and is 0 if no flooding is detected, and the coordinates of the drain tube bounding box $B_{d}$, showing the region of the input image containing the drain tube.

**Stage 2: Temporal Model** The design concept and principle of the second stage is based on our observation that the occurrence of flooding is a time-series event. When the flow of the test chemicals in the spectrometer is obstructed, the organic fluid starts to accumulate at the bottom of the spray chamber and flooding occurs. One obvious clue is the bubbles in the drain tube region stop moving. When the bubbles in the drain tube start to move again, flooding diminishes.

If we input a series of photos into the model, the labels generated should be consistent over time, like 0000111110000 where 0 means no flooding and 1 means there is flooding. With only Mask RCNN, the predicted labels are inconsistent, such as 0010011010101. If we use the relationship between consecutive images, we should be able to improve the accuracy of the model. In other words, the flooding detection is formulated as a sequence classification problem. The commonly used sequence classification techniques [38] includes feature selection based classification, computing similarities between different sequences and statistical models. One way to achieve this is by using Mask RCNN with LSTM. However, a large overhead would be added to the framework and the inference time of the model becomes longer.

Another intuition is the relationship between flooding and the bubbles in the drain tube. Flooding happens when the liquid inside the spray chamber stops leaving the spray chamber. So whenever flooding occurs, the bubbles in the drain tube stop moving. When the bubbles start moving again, the flooding will disappear after some time. As a result, it would be helpful to also detect the bubbles. However, the bubbles are small and transparent objects. They are hard to be detected accurately. One problem is that the air between two bubbles is likely to be predicted as a bubble. So simply using Mask RCNN to detect the bubbles as well as flooding is not enough.

For the input of the temporal model, the confidence scores $S_{f}$ generated from the Mask RCNN are used. We define a window size *w*, which is 10 in our case. For an image at time *t*, $S_{f}$ from time t−w to t+w are combined into a list. So the input length is 2w+1. At the same time, we extract the drain tube region from all the images from t−w to t+w using $B_{d}$ detected at time t=1. The reason is the position of the camera used to take the photos is fixed. By only using the coordinates of the drain tube $B_{d}$ at time t=1, the size of the drain tube region cropped from each image is the same. Then mean squared error (MSE) between two consecutive images is computed to represent whether the bubbles in the drain tube move or not. The MSE follows a certain pattern and gives clues about the flooding. We concatenate the MSE with the confidence scores $S_{f}$ and input them into the temporal model. The temporal model is composed of one dense block followed by a fully connected layer. The dense block has five layers with skip connections between them. The five layers have sizes of 20, 40, 80, 40, 20 respectively. The model is trained for 50 epochs and the batch size is 128. The output of the model is the existence of flooding in the image at time *t*.

### 3.2. Bead Detection

**Figure 5:**
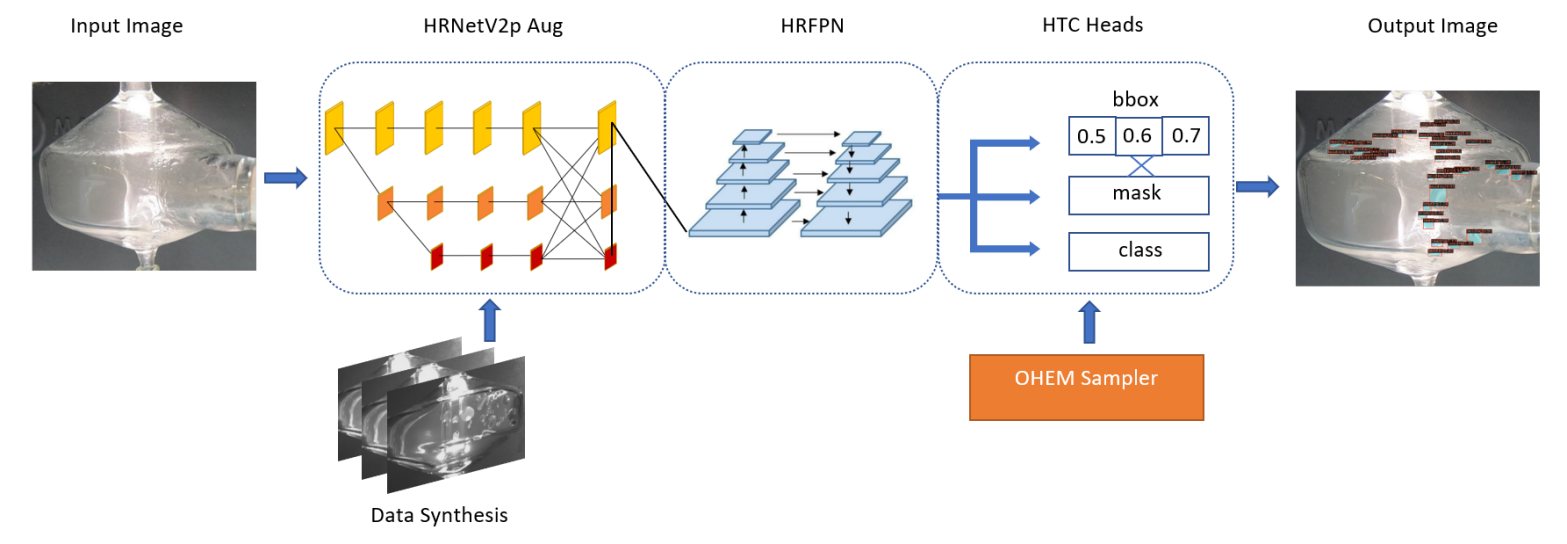
The beading detection pipeline with each component labeled.

Bead detection describes the task of detecting the fluid beading formed on the surface of the spray chamber, via increase in surface tension, in Figure 1b. In this problem, the beads are small objects, transparent and deformable. Additionally, they can appear in dense, localized clusters. We develop a detection pipeline, as shown in Figure 5, to deal with these issues, by optimizing for data augmentation, backbone, detector architecture, and hyperparameter selection.

**Backbone** The selection of backbones is based on their applicability to the small-object detection problem. This involves having feature maps using a kernel suitable for preserving small objects, handling scale invariance, and preserving resolution in these small-scale feature maps. To compare backbones, we choose ResNeXt-101 as the baseline as it precedes most of the succeeding backbone networks (ordered from oldest to newest) and consistently outperforms its ResNet predecessor [39]. Res2Net [40] is a network that deals with multi-scale feature maps by using multiple receptive field sizes at granular levels of an image rather than resolution. TridentNet [41] instead uses a parallel multi-branch architecture, where each branch uses a different receptive field and is then only trained on instances of selective scales, i.e. scale-aware training. ResNeSt is a different approach to scale, using channel-wise attention on different network branches to improve cross-scale information and representation [42]. HRNetV2p is the object detection version of the High-Resolution Network, which works to maintain the same resolution of an image in parallel with convolved branches to identify high-quality representations [9]. Finally, the Swin Transformer is a hierarchical vision backbone that uses a novel shifted-window approach with feature hierarchies to perform object detection and held the object detector SOTA title upon release in March 2021 [43]. The HRNetV2p backbone is selected for beading detection as it can preserve high-quality representations of the beads in its parallel streams and handles scale variance well.

**Detector Architecture** In the beading pipeline, the backbone feeds into the detector. In this case, the architecture includes the neck and head(s), as well as the sampling and loss functions. To obtain the best performance in detecting the small and deformable beads, the choice of architecture is critical. Firstly, a selection of One-Stage detectors in RetinaNet [44], SSD [22] and YOLOv3 [28] were tested for their performance, and then followed by Two-Stage detectors starting with Faster R-CNN [18] and building towards cascaded models in Cascade R-CNN [45] to those including a masking instance branch, to Hybrid Task Cascade (HTC) [10]. As the One-Stage models inherently deal with features differently from a Two-Stage model, they were tested with their respective default backbones. A modified HTC detector is chosen for the beading task which includes three heads of increasing quality and masking branches that are included in the loss calculation for regression.

As the chosen backbone is HRNetV2p, the neck of the bead detector utilizes the High Resolution Feature Pyramids (HRFPN) neck structure. This extends on the original HRNet, and improves object representation by aggregating them from both the upsampled parallel branches - producing higher quality representations than only using the high-resolution stream alone [46].

The Hybrid Task Cascade detector draws inspiration from the high-quality detection from Cascade Mask R-CNN, via the cascade of three localization heads with increasing IoU thresholds of 0.5, 0.6, and 0.7 as in the original paper [10,47]. However, it uses the mask head in combination with the bounding box heads to supplement information flow between them and increases performance by this technique. After conducting tests on altering the IoU thresholds of these heads, as well as their number, the original set was used for best results.

**Hard Negative Mining** The sampler of the detector determines what images are used throughout training based on positive and negative samples. A common example would be a random sampler, which does not take into account any of this information and instead trains in a way to represent the dataset evenly. In this case, the sampler can be used as an important tool for addressing hard example misrepresentation. From investigating inferences on un-cropped chamber images, there are many false positives from the model. Based on this, a negative sampling approach is taken to reduce this by including training examples that do not contain any beading (and only the surrounding scene), to get the model to learn from these mistakes. The Online Hard Example Mining (OHEM) sampler is chosen for this task to improve results significantly [11].

**Figure 6:**
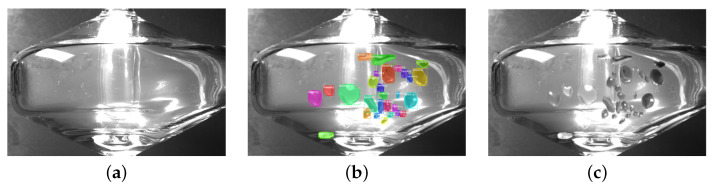
Cut-Paste data synthesis using clean spray-chamber (**a**) and beading instances from an (un-pictured) image coloured in (**b**). The resulting synthetic images in (**c**) are then added to the training set.

**Data Synthesis and Augmentation** One issue with research into the beading phenomena is that the dataset is self-sourced and hand-labeled with mask and bounding box annotations. Since the beading is dense in the spray-chamber region of an image, this is a costly data collection process, resulting in a small dataset of under 100 images, and making it difficult for similar projects. As such, it is anticipated that adding some form of data augmentation or synthesis will aid the bead detector by providing more varied examples, and work to decrease any extreme overfitting. The detector is supplemented by adding3× the number of existing images in the training set as synthetic images, composed of copy-pasted augmented beads from the training set onto augmented clean ‘scenes’ (which denote clear spray-chambers). The beads are augmented with the albumentations [48] library and then superimposed on a clear spray-chamber, chosen at random, which itself undergoes augmentation to change lighting conditions. This is an extension on the Cut-Paste augmentation of [49,50], which saw a competitive increase in performance for detectors trained on synthetic and real data combined when using object masks and augmentation [12]. As in [49], the implemented method uses Gaussian blur to blend the pasted beads onto the background scene to avoid having the model train on pixel artifacts instead of the beads themselves. The synthesized data is shown in Figure 6.

## 4. Dataset and Instruments

Our framework is built for detecting incidents, beads, and flooding, in a controlled industrial environment. Incidents (beads and flooding) are generated manually in a laboratory. We collect the data using two types of cameras, GoPro7 and Basler acA2440. The GoPro7 camera is a small, handheld, and waterproof camera that can take RGB images with 8-bit depth. On the other hand, the Basler acA2440 camera take black and white images with 12-bit depth.

**Figure 7:**
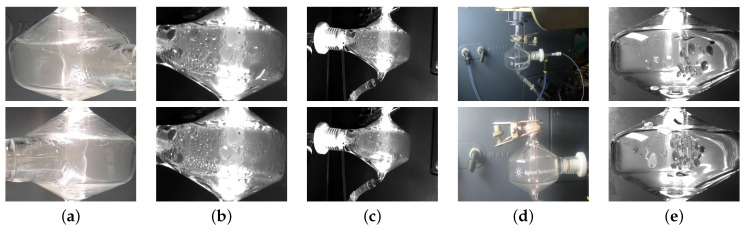
Dataset Types, which are cumulative. (**a**) Type 2, which is cropped of view 2 (GoPro feed). (**b**) Type 3, including cropped of view 3 (Basler feed). (**c**) Type 4, which includes uncropped Basler feed. (**d**) Type 5, which included non-beading, uncropped chambers. (**e**) Type 6, which includes synthetic images.

The dataset for bead detection is collected using a GoPro7 camera from different angles and under different light conditions. The data captured by the camera are videos. Then, random images are sampled from the whole dataset. The whole dataset is composed of 401 images. The classes include spray chamber, nebulizer, drain tube, and beading. Examples of the dataset is shown in Figure 7.

For the beading task, images are taken from two video feeds; one from the RGB GoPro7 and another from Basler acA2440. A total of 50 and 20 images are hand-labeled with mask and bounding-box annotations for beading, from both, respectively. This results in 2041 individual annotations. To conduct the negative mining testing for the beading set, it is necessary to add images to the training set that contain no beading instances at all. To do this, 21 images of clean chambers from three separate settings are annotated with their nebulizer only. This is since those images have no beading present and need to use a redundant class (nebulizer) to be included in training at all. For the beading image synthesis, the synthesizer creates 3× the annotated dataset with images of clean (cropped) chambers with beading instances pasted and gaussian-blended into them. Both the chambers and the beading instances undergo separate and random albumentations. This results in 6130 beading instances in the augmentation set that is mixed with the Type 3 dataset training set, creating the Type 4 training set.

Our method is coded in Python and implemented using the mmdetection framework [51]. We use the PyTorch library to build all of our deep learning models [52]. The computing resources required is a Nvidia Tesla K80 gpu.

The dataset for flooding detection is collected using Basler acA2440. The images are from three consecutive runs and are black and white. 331 images are picked from the whole dataset and labelled. The training set contains 228 images, among which 114 contain flooding. The validation set contains 103 images, and 55 of them contain flooding. The tags include spray chamber, drain tube, flooding, and bubble.

## 5. Experiments

We evaluate all models in the proposed framework using the standard object detection metrics: Average Precision (AP) [53]. The AP is calculated by calculating the precision of the model at different Intersection over Union (IoU) thresholds. For bead detection, we follow the standard COCO evaluation merics and report mean AP (mAP), which is the mean of the AP of different object classes, AP at different IoU thresholds (AP${}_{30}$, AP${}_{50}$, AP${}_{75}$), AP of small objects (AP${}_{s}$), and AP of medium-sized objects (AP${}_{m}$). Among all the metrics, mAP is the most important one as it shows the robustness of the model. AP${}_{30}$ or AP${}_{50}$ shows a more representative performance of the model in real life since when using the model, a specific IoU threshold is chosen to get the final detection results. The IOU [14,54] threshold for the AP is set at different thresholds, 0.3 for beads and 0.5 for flooding. We choose a lower IoU threshold for beads because they are of significantly smaller sizes - as such, minor detection errors can result in a huge performance drop in terms of IoU. We also use precision, recall, and F1 score to evaluate the results. We analyze the results from the flooding detection model and the bead detecting model respectively. We do the ablation studies on our collected dataset, investigating the effect of the temporal model in the flooding detection model.

### 5.1. Flooding Detection

#### 5.1.1. Quantitative Analysis

We show the results of the flooding detection task in Table 1. We compared the performances of the three models, Mask-RCNN, the temporal model with only frame pixel value differences, and the temporal model with both frame pixel value differences and confidence scores of MaskRCNN detection results to demonstrate that the rationale of the model design for this task. One can see that the proposed hybrid method achieves the best performance among all comparing methods in detecting flood incidents. The proposed model (TML) achieves the best performance.

We ablate different parts of the flooding detection model to show their effectiveness. Their performance is shown in Table 1. Only the AP of the Mask RCNN model is shown because the temporal model predicts the existence of flooding instead of object detection. The table shows that with only the Mask RCNN model, the AP is 0.603. The Mask RCNN alone is not enough to predict flooding accurately.

If we only use the temporal model with MSE between frames as input, the precision is 0.941, and the recall is 0.963. However, the performance is still worse than that of the final flooding detection model. The precision is 4.5% lower, and the recall is 3% lower. To find the location of the flooding, the Mask RCNN model is necessary.

The final model we use is the Mask RCNN model followed by the temporal model using both MSE and confidence scores as the input. The model has the highest performance scores, with a precision of 0.985 and a recall of 0.993. This shows the power of our model. We use Mask RCNN for generating bounding boxes and a temporal model for improving the prediction accuracy. If the Mask RCNN fails to find a bounding box while the temporal model predicts the existence of flooding, the bounding box would be the average between the two adjacent frames.

#### 5.1.2. Qualitative Analysis

**Figure 8:**
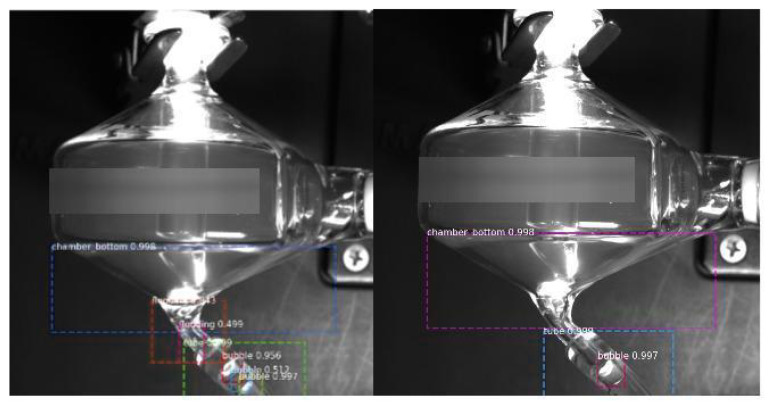
The bounding boxes generated by the flooding detection model. The image on the left side contains flooding, while the image on the right side does not contain flooding.

**Figure 9:**
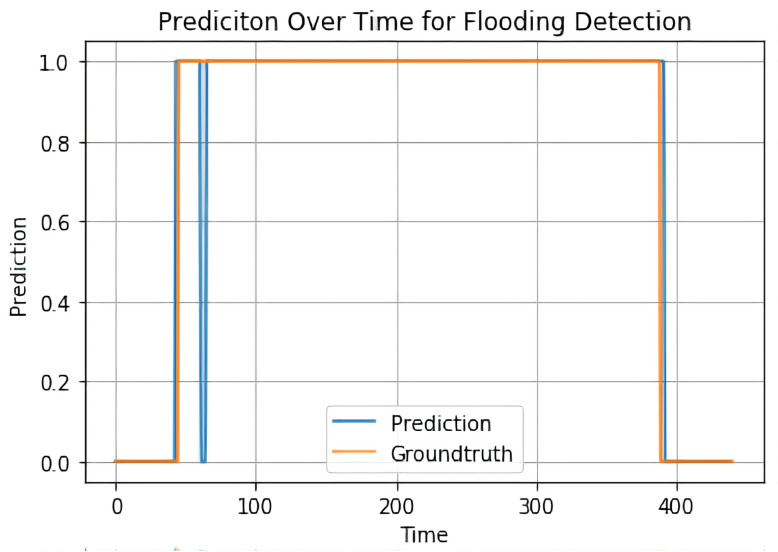
The final prediction of the flooding detection model. 1 means there is flooding while 0 means there is no flooding.

First, we check the bounding boxes generated by the Mask RCNN model. In Figure 8, the results are shown. We can see that the Mask RCNN model can accurately predict the bounding boxes for flooding, the bottom part of the spray chamber, the drain tube region, and the bubbles. Then in Figure 9, we show the final prediction labels of the whole flooding detection model. The blue line represents the predicted labels, while the orange line represents the ground truth labels. We can see that the blue line is close to the orange line, meaning the model can detect the flooding correctly most of the time.

### 5.2. Bead Detection

The precision and recall score of all the models are calculated using a class confidence score threshold of 0.5. We use an IoU threshold of 0.3 for bead detection, based on [50], an evaluation of the average IoU for small objects across the MS-COCO dataset. Objects classed as small in this baseline dataset were found to have an average maximum IoU of 0.29. Therefore, in this paper, IoU of 0.3 is used as the testing threshold and included in all subsequent AP results. Furthermore, with an analysis of the Type 2 dataset, there were no objects in the ‘large’ class, hence the results show up to $AP_{m}$ throughout. To compare each backbone in a controlled environment, each was tested with the Cascade Mask R-CNN detector, with 28 epochs at a learning rate of 0.02, using Stochastic Gradient Descent optimization with 0.9 momentum and 0.0001 weight decay (defaults for mmdetection). This only exception is DarkNet, which is used for the One-Stage YOLOv3 detector and included here for comparison. Based on the results of this backbone testing in Table 2, we choose HRNetV2p as the backbone. This serves as the control factor for testing different detectors in Table 3.

#### 5.2.1. Quantitative Analysis

The main result of the backbone testing is shown in Table 2. As can be seen from the results, the HRNetV2p backbone excels over the others in this task, closely followed by the ResNeSt implementation. However, HRNetV2p outperforms ResNeSt in the $AP_{75}$ category and higher, which is important for a high-quality detector. Hence, it is most appropriate for the beading detection pipeline. The main result of the detector testing is shown in Table 3. What can be seen is that the One-Stage detectors struggle to be competitive with the Two-Stage models in terms of mAP, with the exception of YOLOv3, which surpasses the results of Faster R-CNN.

**Figure 10:**
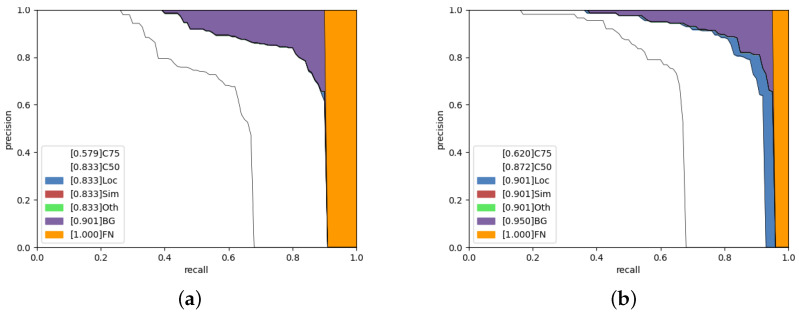
(**a**) HRNetV2p-HTC without OHEM, and (**b**) included. Small Objects are below 32×32 pixels. The area of the graph filled represents the proportion of the results for a given category.

Additionally, the PR curves are shown in Figure 10 below. In this figure, the categories in the legend are shown with their respective Area-Under-Curve. These categories are modified by the COCO team in their evaluation API, from the original Derek PR curve implementation [58,59]. For example, the middle black line between C75 and C50 represents the change from using IoU 75% vs. 50%, giving an increase in mAP from 0.620 to 0.872, respectively, for (b). Next, **LOC** is localization error with IoU set to 0.1; this describes the errors that occur when reducing the IoU even further from 0.5 to 0.1. **Sim** is superclass misclassification. **Oth** is after removal of class confusion, **BG** is background misclassification and finally **FN** is False Negatives. These outputs show that the largest cause of error in the model is from background misclassification and false negatives. When using hard-negative mining, we can see that the proportion of False Negatives (orange area), as well as background misclassification (purple), decreases. However, there is an increase in localization error; likely due to the increase in varied images without beads present to prevent any overfitting.

#### 5.2.2. Qualitative Analysis

**Figure 11:**
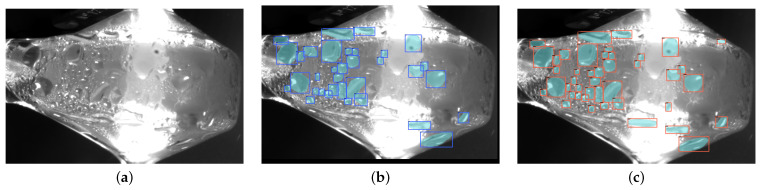
The results of the beading detection pipeline on a Basler image. The input image (**a**), with ground truth labels (**b**), shows inference results in (**c**).

The resulting images of bead detection are shown in Figure 11. It can be noted that the beading detector can correctly identify beads that correspond to the Ground-Truth labels, as well as having labeled some additional beading instances that were not picked up in the hand-labeling process. This confirms that the detector can find these beading instances with high accuracy, while also highlighting the importance of proper labeling and the need for a larger dataset to learn. This motivates the use of the augmented synthesis pre-training implemented in the final model.

**Figure 12:**
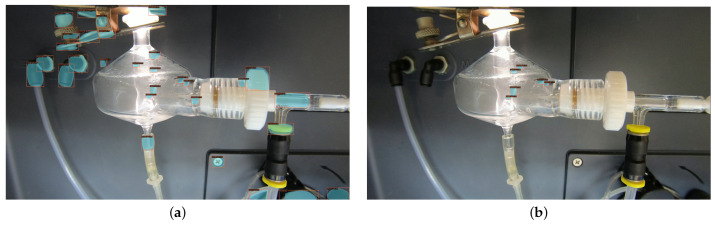
Inference on an un-cropped chamber, when model is trained without negative mining (**a**), and when trained with the OHEM sampler (**b**).

Further experiments were conducted on un-cropped views of the chamber with beading present to test the theory that the model may overfit and become over-confident on its specific chamber view, which can ultimately hurt performance. The model was tested with and without the OHEM sampler, and the results are displayed in Figure 12. Without training on negative samples, it is clear that the model is looking for round objects indiscriminately, and labels circular shapes outside of the chamber with high confidence. This qualitatively verifies the importance of the negative mining usage in the beading pipeline, to remove the high number of false negatives. While the use case of the model will be on cropped video footage in one pose and lighting, it is important to teach the model the fundamentals of the beading instances rather than be in-capable once the pose is slightly altered.

#### 5.2.3. Ablation Study

We ablate different parts of the beading pipeline to assess their effectiveness, similar to the flooding model. The results are shown in Table 4. Specifically, we focus on the additions of the hard-negative mining approach via OHEM, and the data synthesis pre-training (denoted by Aug. herein). The implementation of HTC with the HRNetV2p backbone is shown for reference.

### 5.3. Inference Speed Analysis

Our malfunction detection model can detect four frames per second (fps). The temporal flooding detection model can also process four fps. Even though the temporal flooding detection model requires a series of results (21 frames) as input, the model can make predictions consistently once 5 s have passed. In reality, an experiment using the spectrometer usually lasts for hours. Being able to process four frames per second is enough to report any malfunctions detected.

### 5.4. Discussion

Based on the experiments, we can conclude that the framework proposed is capable of detecting spectrometer incidents under controlled environments. The flooding detection model demonstrates the effectiveness of adding time series data with heuristics based on features of the specific incident. By adding an extra temporal model to the deep learning object detection model, there is a huge increase in both accuracy and recall. On the other hand, the beading detection uses a Two-Stage detection pipeline with synthetic data augmentation and negative mining to reach an mAP of 83.9% on fixed-pose spray chambers. The use of a high-resolution backbone with FPN, as well as the HTC detector and the previously mentioned augmentations were able to achieve much higher accuracy than a naive approach.

The implementation of our pipeline is simple in the real-world scenario. A fixed-position camera is required to collect data and send the data to a cloud server or a computer synchronously. Since the locations of the camera and the spectrometer are expected to be fixed during the run of one experiment, the model only needs to detect the location of the spray chamber, nebulizer, and drain tube once at the beginning of the run or every few minutes. Then the cropped spray chamber region images and cropped drain tube region images are sent to corresponding object detection models to generate the result. The pipeline can process four images every second, which is sufficient to generate warning of any detected malfunctions.

## 6. Conclusions

We present an end-to-end framework for detecting incidents on spectrometers in an industrial environment. The first type of incident to be detected is the flooding of the spray chamber and is visualized as the accumulation of liquids beyond the safe level. Based on the qualitative result, identifying the flooding phenomenon only relies on a single image is challenging and difficult. The temporal information increases the performance of the flooding detection on a single image by better describing the time-series property of the flooding incident and representing the relationship between flooding and bubbles in the drain tube. The second type of incident is the transparent and deformable beads on the surface of the spray chamber. One challenge of bead detection is to design a suitable backbone to handle the scale invariance and preserve feature maps for small objects. The backbone HRNetV2p with the High-Resolution Feature Pyramids is selected to generate higher quality object representation using both the upsampled parallel branches than using only the high-resolution stream alone. The ablation study of the bead detection pipeline demonstrates the effectiveness of the OHEM sampler and the synthetic data augmentation. The framework achieves nearly perfect accuracy for flooding detection and a reasonably good result for bead detection.

In addition to the main findings by the autonomous incident detection framework, there are also some limitations of our work. First of all, due to the characteristics of transparent objects, the detection of incidents is affected by the light source in the image. The reflection of lights at the surface of the spray chambers and drain tubes makes detection challenging. One potential solution is to include training images captured from different lighting sources to ensure different lighting intensities are included. The stimulation of lighting using the contrast adjustment as the data augmentation can be applied to the current pipeline. Secondly, the angle of the camera affects the performance of our pipeline. Currently, all data collected are taken by a camera from the front of the spectrometer. In reality, the camera may be attached to the top of the spectrometer. Whether the model can perform well under this unseen circumstance is unknown. Cameras will be located at different positions to ensure diversities of bead detection images. In other words, the data labeling process could be extended to many more spray-chamber images, from different poses as well, to improve the training process and increase the robustness of the model to transfer to different views. If the 3D CAD model for the spray chamber and the depth information of each component in the bead detection scene can be obtained in the future, the extra depth image as an additional feature map will be added to improve the robustness of bead detection. Finally, the model performance on an external dataset can be assessed. This would test the generalizability of the pipeline to small object classes in general and not just one class for a specialized case.

Our research has broader implications. Despite the fact that we only tested our method on detecting beads and flooding in spectrometers, it can be used in other situations. We have shown that deep learning models are effective in detecting beads and fluids. There are many glass laboratory apparatus working with liquids in laboratories. If a long-running experiment is required, our technique can automatically detect the occurrence of incidents. Furthermore, by detecting the bubbles, similar to beads, in tubes with high accuracy, we can track the bubbles and calculate the speed of the flow. Even though liquid flow sensors can compute the speed of the flow, they require installment and integration into the devices, our method only requires a camera with no contact with the corrosive samples. The speed of the liquid flow in the device is important since a lower speed may imply damage or aging of the pipes/tubes. Another research plan we have is to detect other incidents, such as leakage at conjunction points or wrong pump pressure. This shows another advantage of using deep learning models for detecting multiple incidents, as long as the incidents can be identified by visual information only, and different detection models can be trained and integrated into one single malfunction detection system.

## Figures and Tables

**Table 1:** Final Results of flood detection. MR means Mask RCNN model. TM means temporal model using only mean squared error (MSE) between drain tube region frames as input. TML means using both MSE between consecutive frames and the predicted bounding boxes confidence scores from Mask RCNN. P, R, and F1 stand for precision, recall, and f1 score, respectively.

MR	TM	TML	AP	P	R	F1
✔			0.603	0.885	0.750	0.812
	✔			0.941	0.963	0.952
✔		✔		0.986	0.988	0.987

**Table 2:** Backbone testing results. The Cascade Mask R-CNN detector is used except for DarkNet, which is native to the YOLOv3 detector. The best result in each column is in bold.

Backbone	mAP	AP_30_	AP_50_	AP_75_	AP_*s*_	AP_*m*_
DarkNet [28]	62.1	86.7	86.5	66.6	58.1	68.9
x101 64-4d [55]	60.5	84.9	82.9	55.1	47.2	66.8
RegNet [56]	59.0	85.5	82.2	52.8	49.2	64.1
ResNeSt [42]	64.7	89.9	88.0	59.7	56.7	69.4
Res2Net [40]	58.7	83.3	80.4	53.1	46.3	64.8
Swin-96 [43]	56.3	82.4	78.9	46.5	49.3	60.1
TridentNet [41]	42.2	76.6	67.3	19.2	37.7	44.9
HRNetV2p [9]	**69.6**	**90.1**	**89.0**	**71.7**	**59.1**	**74.8**

**Table 3:** Detector testing results. The two-stage detectors use the HRNetV2p backbone. The single-stage detectors denoted with * use their respective backbones. Proposed denotes HTC with OHEM sampler and augmentation training. The best result in each column is in bold.

Detector	mAP	AP_30_	AP_50_	AP_75_	AP_*s*_	AP_*m*_
SSD-512 * [22]	27.2	59.8	46.9	7.50	9.70	36.8
RetinaNet * [44]	42.2	76.6	67.3	19.2	37.7	44.9
YOLOv3 * [28]	62.1	86.7	86.5	66.6	58.1	68.9
YOLOF * [57]	56.4	85.5	78.8	49.9	49	61.1
Faster [18]	56.3	82.4	78.9	46.5	49.3	60.1
Libra [19]	66.0	87.2	86.6	69.3	56.0	70.9
Cascade [45]	69.3	91.2	90.4	69.9	60.6	73.6
Cascade Mask [47]	69.6	90.1	89.0	71.7	59.1	74.8
HTC [10]	70.3	90.9	90.1	70.9	61.3	**74.8**
Proposed	**83.9**	**96.0**	**95.1**	**84.7**	**65.6**	71.4

**Table 4:** Beading detection ablation results.

HTC	OHEM	Aug.	mAP	AP_30_	AP_50_	AP_75_
✔			70.3	91.3	90.1	61.3
✔	✔		80.4	95.8	94.9	82.6
✔	✔	✔	83.9	96.0	95.1	84.7

## Data Availability

Not applicable.

## Abbreviations

The following abbreviations are used in this manuscript:

AP	Average Precision
BG	Background Misclassification
MSE	Mean Square Error
OHDM	Hard-Negative Mining
HRFPN	High Resolution Feature Pyramids
HTC	Hybrid Task Cascade
IoU	Intersection over Union
R-CNN	Region-based Convolutional Neural Network
